# *In vitro* and *in vivo* datasets of topically applied ketorolac tromethamine in aqueous humor using Raman spectroscopy

**DOI:** 10.1016/j.dib.2019.104694

**Published:** 2019-10-22

**Authors:** Shuo Zhang, Christian J.F. Bertens, Roel J. Erckens, Frank J.H.M. van den Biggelaar, Tos T.J.M. Berendschot, Carroll A.B. Webers, Rudy M.M.A. Nuijts, Marlies Gijs

**Affiliations:** a University Eye Clinic Maastricht, Maastricht University Medical Center+, P. Debyelaan 25, P.O. Box 5800, 6202, AZ, Maastricht, the Netherlands; b Maastricht University, School for Mental Health and Neuroscience, University Eye Clinic Maastricht, Universiteitssingel 50, P.O. Box 616, 6200, MD, Maastricht, the Netherlands; c Chemelot Institute for Science and Technology (InSciTe), Urmonderbaan 20F, 6167, RD, Geleen, the Netherlands; d Department of Ophthalmology, Zuyderland Medical Center, Heerlen, the Netherlands

**Keywords:** Raman spectroscopy, Ophthalmology, Ketorolac tromethamine, Rabbits, Aqueous humor, Pig eyes

## Abstract

This article includes datasets acquired by Raman spectroscopy from *in vivo* and *in vitro* ocular samples collected from the dataset from Bertens and Zhang *et* *al.*, “Confocal Raman spectroscopy: Evaluation of a non-invasive technique for the detection of topically applied ketorolac tromethamine *in vitro* and *in vivo*” (Bertens and Zhang, *et al.*). Detection of ketorolac tromethamine in pig eyes was performed *in vitro* and rabbit eyes *in vivo*. Extracted aqueous humor samples from pig and rabbit eyes were measured *in vitro* using a cuvette. This manuscript shows the spectral Raman data without pre-treatment or analysis from ocular tissues and provides further information towards aqueous humor research via alternative data processing methods. Furthermore, the raw data enclosed may be used for future aqueous humor investigations and pharmaceutical research.

**Table:** Specifications Table

Subject	Pharmacology, Toxicology and Pharmaceutics (General)
Specific subject area	Raman spectroscopy of pharmaceutical compounds detection.
Type of data	Figures Spectra Tables
How data were acquired	Raman spectra were recorded with a high-performance Raman module model 2500, River Diagnostics®, Rotterdam, the Netherlands.
Data format	Raw unanalyzed Raman spectra
Parameters for data collection	A 785 nm, 26 mW continues diode laser (Innovative Photonic Solutions SM 785 nm), and a 671 nm, 14 mW continues diode laser (Laser Quantum Ignis 671 and SMD 6000) were used to excite the samples. Raman spectra were collected with 60 seconds exposure time and 3 frames, and 30 seconds exposure time with 2 frames, for the *in vitro* and *in vivo* study, respectively.
Description of data collection	*In vitro* measurements, the enucleated eyes were immersed in ketorolac containing fluid overnight, where after they were fixed in a holder and mounted on an adjustable lens mount. *In vivo* measurement, the rabbits were fixed and anesthetized respectively. In cuvette measurement, the sample were load in a cuvette and fixed with a holder.
Data source location	Maastricht University, Maastricht, The Netherlands
Data accessibility	Data are supplied with the article
Related research article	Christian J.F. Bertens, Shuo Zhang, Roel J. Erckens, Frank J.H.M. van den Biggelaar, Tos T.J.M. Berendschot, Carroll A.B. Webers, Rudy M.M.A. Nuijts, Marlies Gijs. Confocal Raman spectroscopy: evaluation of a non-invasive technique for the detection of topically applied ketorolac tromethamine *in vitro* and *in vivo*, International Journal of Pharmaceutics https://doi.org/10.1016/j.ijpharm.2019.118641

**Table:** 

**Value of the Data** • The dataset could be used for further composition analysis of the aqueous humor and for future pharmaceutical research, to increase sensitivity of Raman systems. • The dataset can be useful for researchers who are interested in the aqueous humor composition, ocular pharmaceutics, Raman spectroscopy, and software engineers. • Alternative processing methods could be applied to exact other compounds in the aqueous humor or to enhance signals. • This dataset offers a large cohort of animals measured on both eyes, 5 times.

## 1 Data

**Fig. 1:**
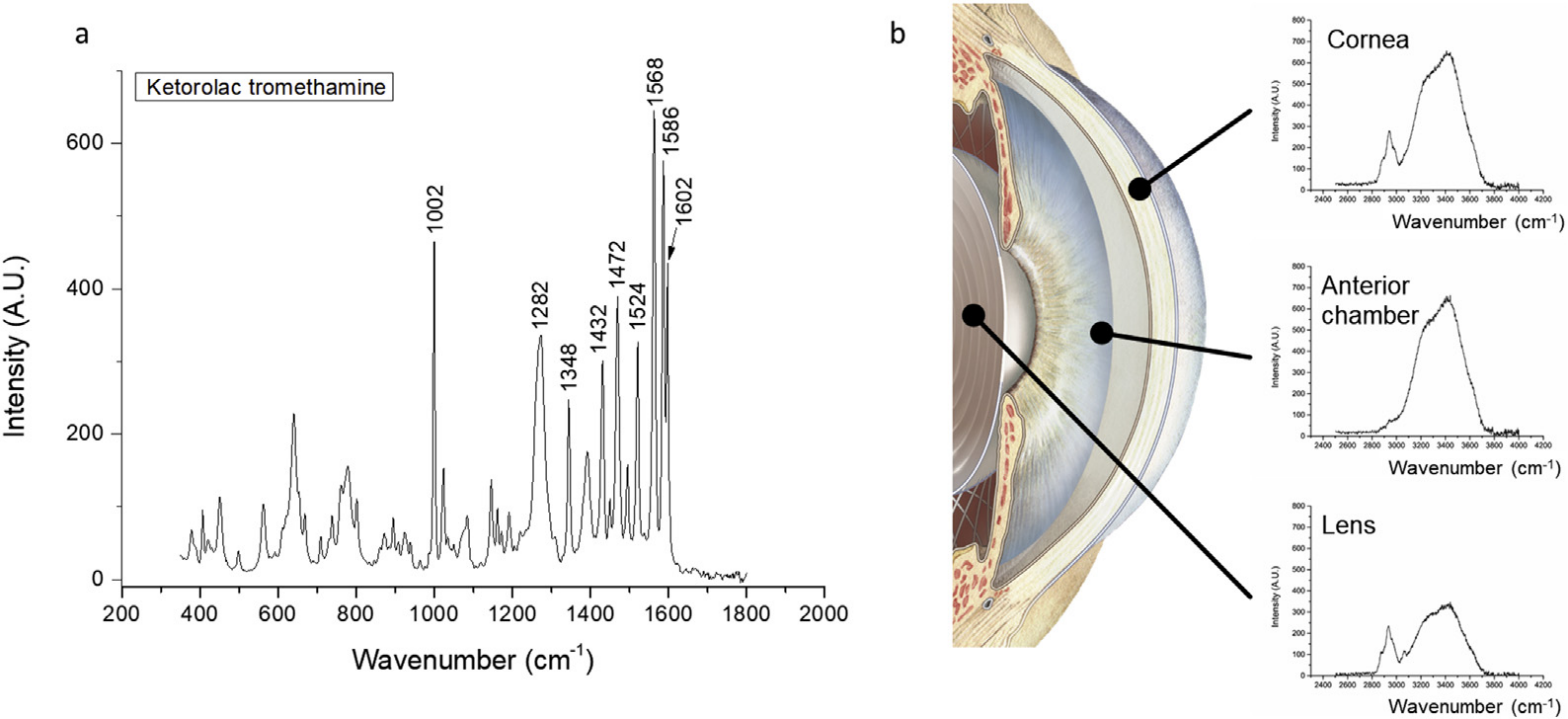
(a) Fingerprint spectra of Ketorolac powder. (b) Determination of the location in the eye using high-wavenumber Raman spectra. Spectra are from pig eyes, 3 frames of 10 seconds averaged measured using a Jena lens.

The data contains unanalyzed Raman spectra obtained from pig eyes (*in vitro*) (1.1, see supplementary files folder ‘*in vitro* pig eyes’ and ‘*in vitro* cuvettes, aqueous humor from pig eyes’), rabbit eyes (*in vivo*) (1.2, see supplementary files folder ‘*in vivo* rabbit eyes’), and aqueous humor samples (*in vitro*, see supplementary files folder ‘*in vitro* cuvettes, aqueous humor from rabbit eyes’) (1.3). Based on the differences of the samples, three types of set-ups were used on each dataset. For pig eye measurements *in vitro*, a long-working-distance microscope objective lens (Jena lens alone or a Gonio lens combined with an f60 lens) was utilized (see supplementary files ‘*in vitro* pig eyes’ folder ‘jena lens’ or ‘gonio’). For the rabbit eyes measurements *in vivo*, a Gonio lens combine with a f60 lens was used. For cuvettes measurements, an f80 lens was used when the sample was measured in a Brand® cuvette [2]. For each experimental set-up, the fingerprint-wavenumber region (patterns specific for a drug-molecule, ranging from 350 cm^−1^ to 1800 cm^−1^) and the high-wavenumber region (higher energy shifted, ranging from 2500 cm^−1^ to 4000 cm^−1^) were included. The fingerprint spectra dataset was used for detection of intraocular ketorolac tromethamine as described in the article of Bertens and Zhang *et* *al.* [1]. Several peaks could be identified in the fingerprint region spectrum of a ketorolac tromethamine sample (Fig. 1a). Only major peaks specific for ketorolac tromethamine were selected. Those peaks are assigned to certain chemical bonds or vibration modes. The assignment of the ketorolac related peaks is presented in Table 1 [1]. Due to the spectrometer's spectral resolution (2 cm^−1^), the peak observed at 1586 cm^−1^ is assigned to NH_2_ deformation [3]. The peak of 1524 cm^−1^ is assigned to in-plane vibrations of the conjugated –C=C-. The observed peak at 1472 cm^−1^ is assigned to C=N stretching and the peak at 1282 cm^−1^ is assigned to CH_2_ wagging vibrations. Because Raman spectrum of the cornea, aqueous humor, and lens show different patterns in the high-wavenumber region, spectra from this region could be used as guide for location determination in the ocular tissue (Fig. 1b) [[4], [5], [6]].

**Table 1:** Main characteristic bands assignment of ketorolac [7].

Peak location (wavenumber)	Intensity	Peak Assignment
1002 cm^−1^	very strong	Phenylalanine or a C–C aromatic ring stretching
1282 cm^−1^	medium	CH_2_ wagging vibrations
1348 cm^−1^	weak	An unassigned mode
1432 cm^−1^	strong	CH bond [5]
1472 cm^−1^	medium	C=N stretching
1524 cm^−1^	medium	In-plane vibrations of the conjugated –C=C-
1568 cm^−1^	very strong	COO^−^
1586 cm^−1^	strong	NH_2_ deformation [3]
1602 cm^−1^	medium	Phenylalanine or a C=C bond.

### 1.1 *In vitro*, dataset

**Fig. 2:**
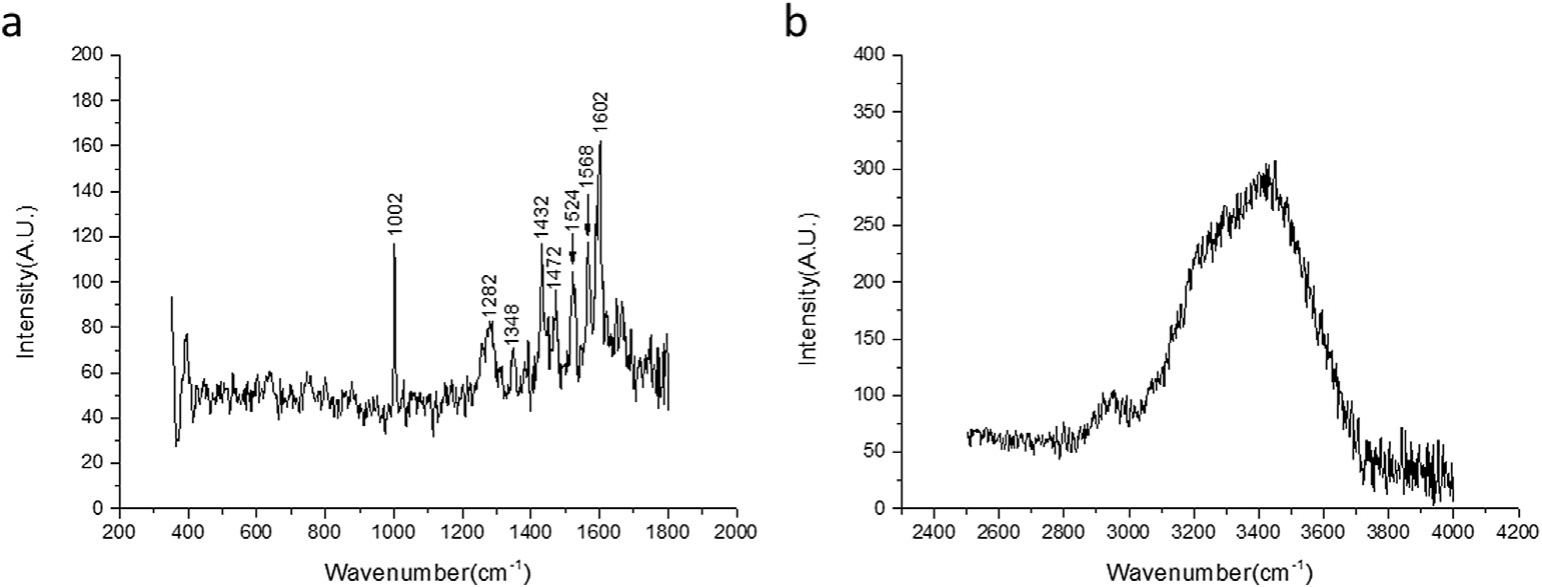
Raman spectrum of a pig eye soaked in a 5% ketorolac solution obtained by Jena lens. (a) Fingerprint spectrum, obtained using 60 seconds and averaged for 3 frames. (b) High-wavenumber spectrum, obtained using 60 seconds and averaged for 3 frames. No correction has been applied on the spectra.

Pig eyes (enucleated) were immersed in the dark at 4 °C for 24 hours *in vitro* in different concentrations of ketorolac solutions (0.05%–5.0%) before the measurements (see supplementary files folder ‘*in vitro* pig eyes’). For each concentration, three eyes were measured by Raman spectroscopy. An example spectrum obtained from a pig eye is shown in Fig. 2. The location in the eye was determined using the high-wavenumber spectra (Fig. 2b).

### 1.2 *In vivo* dataset

**Fig. 3:**
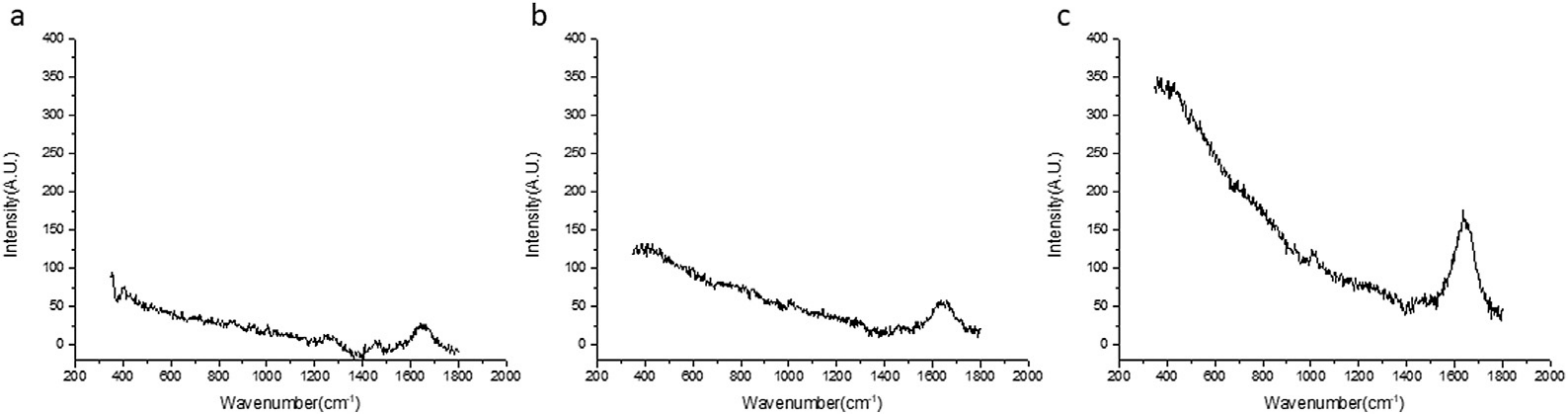
*In vivo* Raman spectrum of the right eye of a rabbit with different integration times, averaged for 2 frames. (a) Shows the graph for 10 seconds, (b) 15 seconds, and (c) 30 seconds.

New-Zealand white rabbits received 50 μL Acular® three times a day in their right eye. At the same time, they received a drop of buffered saline solution (BSS) in their left eye as a control (see supplementary files folder ‘*in vivo* rabbit eyes’). The measurement parameters of the Raman system were optimized using the first four rabbits. Different integration times (10, 15, or 30 seconds) were measured to acquire the optimum Raman signal. The following measurements were performed using an integration time of 30 seconds. During these measurements, hardware influences were observed. Further optimization of the processing method can be seen in Bertens and Zhang *et* *al.* [2]. The difference of the variant integration times can be found in Fig. 3, for example, the spectrum intensity at 400 cm^−1^ is from 74 A .U. with 10 second integration time (Fig. 3a), 127 A .U. with 15 second integration time (Fig. 3b) and 333 A .U. with 30 second integration time (Fig. 3c). Rabbits were measured according to the schedule in Table 2.

**Table 2:** *In vivo* integration time of the Raman measurements of the rabbits.

No.	Name	Day 0	Day 7	Day 14	Day 21	Day 28
1	PLAC	x	10 s	15s	15s	30s
2	PLBT	10s	x	15s	15s	30s
3	PKXF	10s	10s	15s	15s	30s
4	PKYJ	10s	10s	15s	15s	30s
5	PNRS	30s	30s	30s	30s	30s
6	PNPH	30s	x			
7	PNPJ	30s	30s	30s	30s	30s
8	PNLJ	30s	30s	30s	30s	30s
9	POLI	30s	x			
10	POBS	30s	30s	30s	30s	30s
11	PPDI	30s	30s	30s	30s	30s
12	POHI	30s	30s	30s	30s	30s

Integration time is shown in seconds, ‘x’ represents a failed measurement or no data. 2 frames per measurement were used.

### 1.3 *In vitro*, cuvettes dataset

**Fig. 4:**
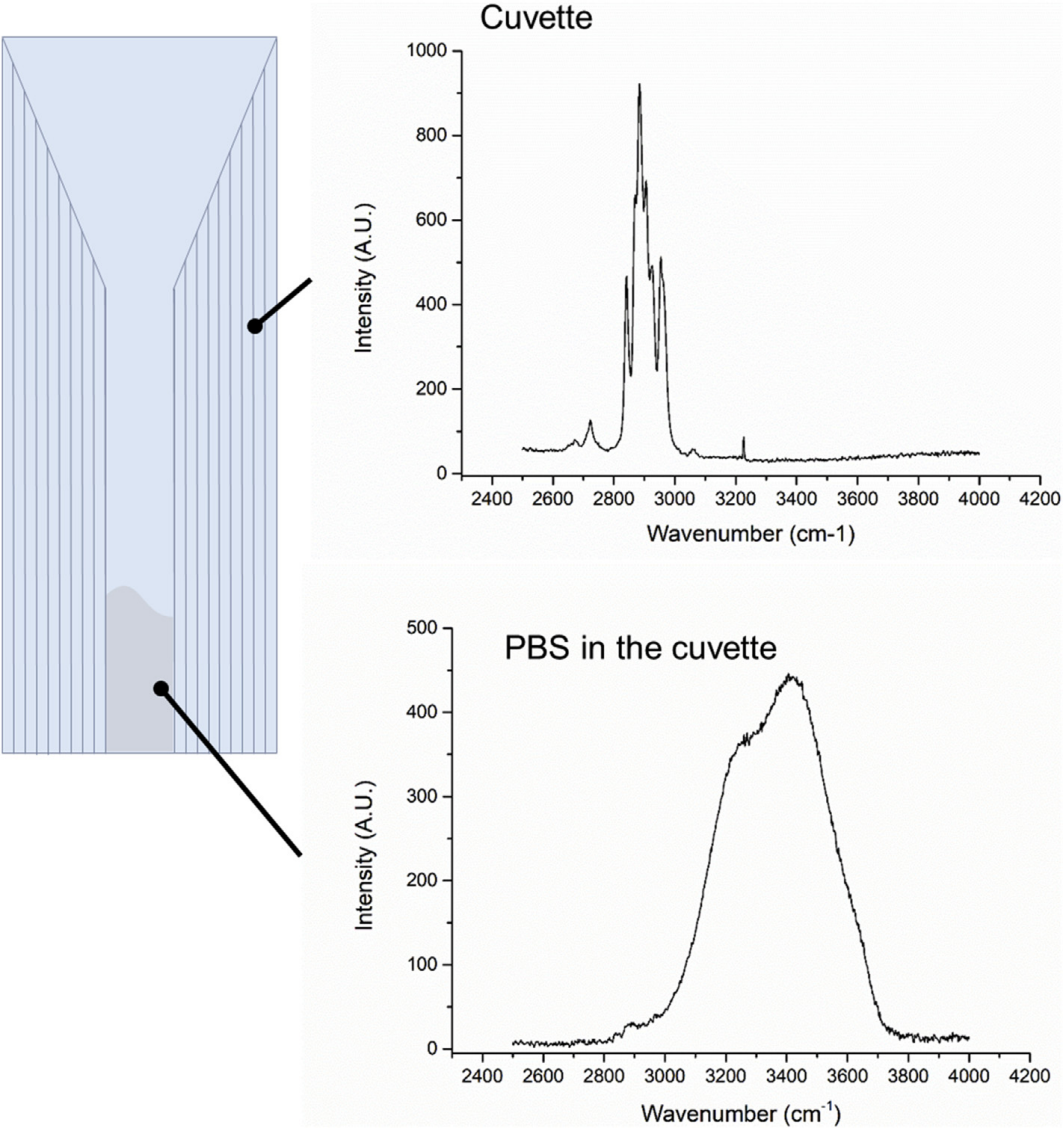
Laser focus positioning in a cuvette filled with PBS (pH 7.4) using the high wavenumber spectrum.

Immediately after intra-ocular Raman measurements (both *in vitro* & *in vivo*), 100 μL–150 μL of aqueous humor was drawn from the pig eyes, and 50 μL was drawn from the right eye of each rabbit. The aqueous humor samples were frozen on dry ice and stored in a −80 °C freezer until use. When used, the location of focus was determined with the high wavenumber spectra, as shown in Fig. 4.

**Fig. 5:**
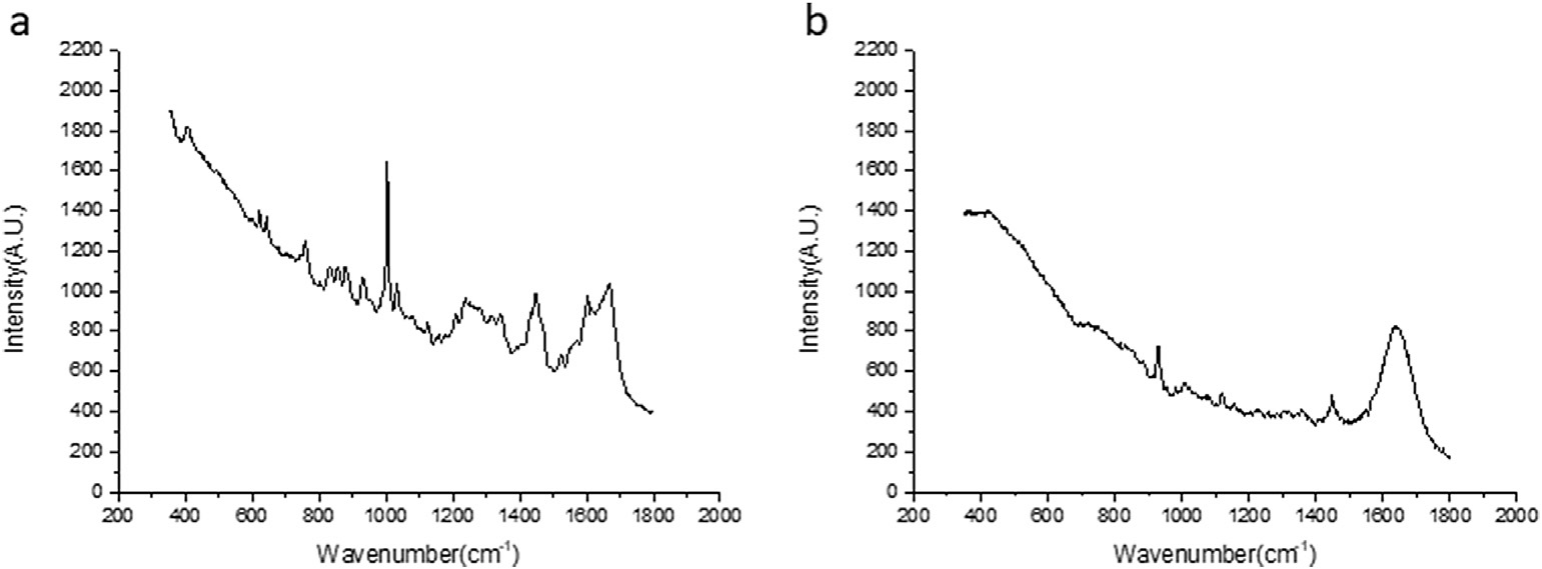
Raman spectrum of aqueous humor samples from, (a) a 0.5% ketorolac submerged pig eye (3 frames of 60 seconds), and (b) from a rabbit eye (3 frames of 60 seconds on pkxf samples).

Fingerprint spectra were collected to determine ketorolac concentrations in the aqueous humor. Spectrum examples of pig and rabbit aqueous humor are show in Fig. 5a and Fig. 5b, respectively (see supplementary files folder ‘*in vitro* cuvettes’). Further background subtraction needs to be applied for analyses.

## 2 Experimental design, materials, and methods

### 2.1 Raman spectroscopy system

Two diode lasers were utilized as an excitation light source for Raman spectroscopy: a 26 mW 785 nm laser (Innovative Photonic Solutions SM 785 nm, Monmouth Junction, NJ, US) or a 14 mW 671 nm laser (Laser Quantum Ignis 671 and SMD 6000, Konstanz, Germany). A high-performance Raman spectrometer module (model 2500, River Diagnostics®, Rotterdam, the Netherlands) was utilized for Raman spectra recordings [8]. A 25 μm diameter pinhole was integrated within the spectrometer for the confocal Ramans spectroscopy detection. An air-cooled charge-coupled device (CCD) camera with operating temperature −60 °C was integrated within the spectrometer for signal detection. The Raman spectrometer is capable of collecting Raman scattering wavenumber ranges in 350 cm^−1^ - 1800 cm^−1^ and 2500 cm^−1^ - 4000 cm^−1^ with 2 cm^−1^ spectral resolution. A diverged laser beam out of the spectrometer is converted to a collimation beam by a lens with focus length of 80 mm (f80). Depending on the measurement, the lens setup was adapted. The system was used in single point modus and location in the sample was determined using the high wave numbers (671 nm laser).

### 2.2 *In vitro* measurement of enucleated pig eyes

**Fig. 6:**
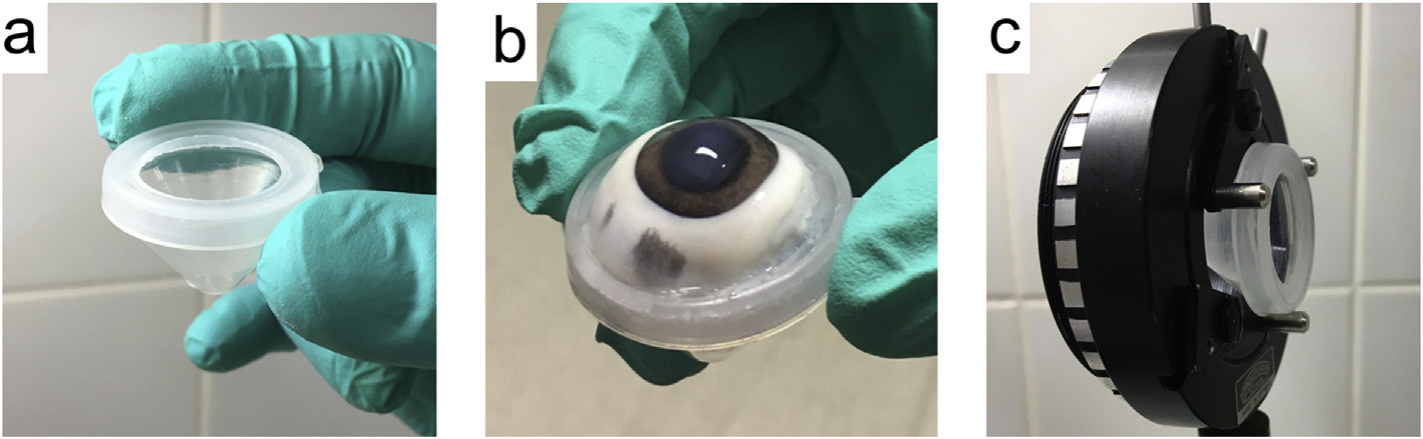
Holder for enucleated eyes. (a) shows an empty holder, (b) shows a holder with a pig eye, and (c) shows the empty holder on an adjustable lens mount.

Fresh domestic pig (*Sus Scrofa Domesticus*) eyes were obtained from a local abattoir (“Slachthuis Kerkrade Holding”, Kerkrade, the Netherlands). The enucleated eyes were transported to the laboratory on ice and used within 3 hours after enucleation. Before use, the pig eyes were inspected with a stereo microscope (Olympus SZX9, Tokyo, Japan). Only eyes with clear corneas without visible corneal damage were used in the experiment. The excess tissues of the eye were removed carefully where after the eyes were washed in phosphate buffered saline (PBS) (pH of 7.4). Meanwhile, ketorolac (MSN laboratories, Telangana, India) was dissolved in PBS creating concentrations of 0.05%, 0.1%, 0.125%, 0.25%, 0.5%, 1.0%, 1.25%, 2.5%, and 5.0%. The pig eyes were submerged in 15 mL of a diluted ketorolac solution. As negative control, PBS was used, and as positive control 0.5% ketorolac ophthalmic solution (Acular®, Allergan, Dublin, Ireland) was used as submerging solution. For each concentration, three eyes were used. Before the Raman measurements, pig eyes were stored in the dark at 4 °C for 24 hours. Before measurements were taken, the eyes were inserted in a home-designed holder (Fig. 6).

**Fig. 7:**
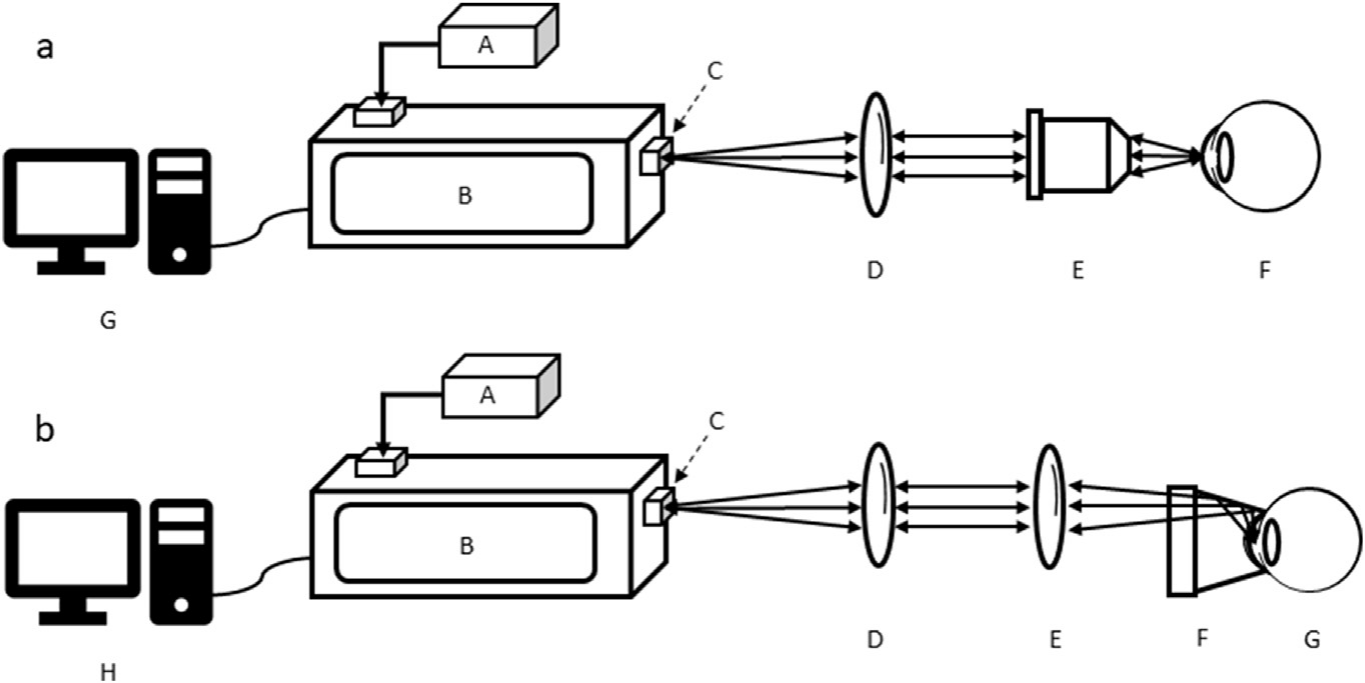
*In vitro* and *in vivo* settings of the Raman system. (a) The set-up is for *in vitro* pig eye measurements by a Jena lens. (A) laser; (B) Raman module, with (C) 25 μm pinhole; (D) collimation f80 lens; (E) objective (Jena lens); (F) pig eye; (G) computer. (b) The set-up is for *in vitro* pig eye and *in vivo* rabbit measurements, a Gonio lens in combination with an f60 focus lens are used for focus in the anterior chamber of the animal eye. (A) laser; (B) Raman module, with (C) 25 μm pinhole; (D) collimation f80 lens; (E) f60 lens; (F) a Gonio (one-mirror) lens; (G) pig eye (*in vitro*) or rabbit eye (*in vivo*); (H) computer. Arrows indicate direction of excitation laser light and backscattered Raman light.

A long-working-distance microscope objective lens (Jena lens, magnification ×25; numerical aperture = 0.50; focal length = 10 mm; Carl Zeiss, Jena, Germany) was used as focus lens for the Raman system (Fig. 7a). A f60 lens combined with a Gonio lens (Haag-Streit Meridian, CGA1, Köniz, Switzerland) also been used for pig eye measurement (Fig. 7b). Methocel® 2% (OmniVision, Santa Clara, CA, US) was used to connect the Gonio lens to the cornea. The samples were exposed to 3 frames for 60 seconds. A detailed description can be found in the manuscript from Bertens and Zhang *et* *al.* [1].

### 2.3 *In vivo* measurement of the rabbit eyes

Twelve New-Zealand white rabbits (weight ranged from 2.0 kg to 2.5 kg upon arrival) were obtained from Envigo (Horst, the Netherlands). The rabbits were group housed with 6 animals per cage with males and females separated. The rabbits had *ad libitum* access to water and food. One week was given to acclimatize before rabbits were used in the experiments. The rabbits were treated with 50 μL Acular® in the lower conjunctival fornix of their right eye. The contralateral eyes were treated with 50 μL sterile buffered saline solution (BSS, B. Braun, Melsungen AG, Germany manufacturer). Both treatments were performed three times a day. Measurements were taken on day 0, day 7, day 14, day 21, and day 28. Four rabbits were used to optimize the system parameters as shown in Table 2.

Rabbits were measured using setup as shown in Fig. 7b. During the examinations, rabbits were anesthetized intramuscularly with ketamine (Alfasan, Woerden, the Netherlands) and midazolam (Actavis, Dublin, Ireland), 50 mg/kg and 5 mg/kg, respectively. Both eyes of the rabbit were measured by the Raman system. All measurements were performed at random, 1–3 hours after receiving the eye drops. Measurement was performed with 30 second exposure times using 2 frames. All animal procedures were conducted according to the ARVO Statement for the Use of Animals in Ophthalmic and Visual Research and the Guidelines of the Central Laboratory Animal Facility of Maastricht University. All protocols were approved by the Central Committee for Animal research and were in accordance with the European Guidelines (2010/63/EU).

### 2.4 *In vitro* measurement of the aqueous humor

For cuvette detection, 50 μL–150 μL aqueous humor was obtained from an anterior chamber paracentesis from the eyes using an insulin syringe (BD Micro-Fine™, Becton Dickinson, NJ, US). 50 μL was drawn from rabbit eyes after topical sedation (1 drop 0.4% Oxybuprocaine hydrochloride solution (Bausch & Lomb Pharma, Brussels, Belgium)), 100 μL–150 μL was drawn from the pig eyes. As a negative control, 100 μL aqueous humor was drawn from seven healthy control rabbits within 10 minutes after sacrifice, no topical treatment nor anaesthetics were used.

**Fig. 8:**
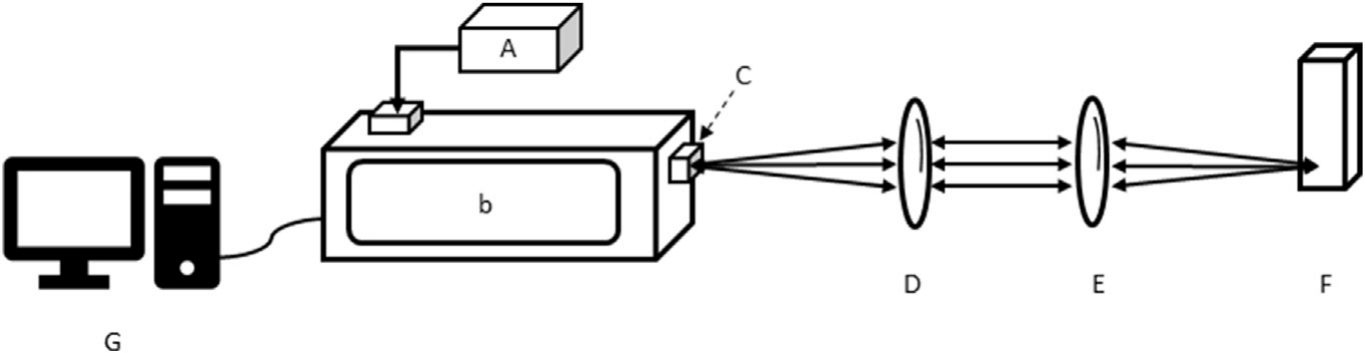
*In vitro* settings of the Raman system with a cuvette. (A) laser; (B) Raman module, with (C) 25μm pinhole; (D) collimation f80 lens; (E) focusing f80 lens (F) samples with in cuvette; (G) computer. Arrows indicate direction of excitation laser light and backscattered Raman light.

All aqueous humor samples were frozen on dry ice immediately after sampling and stored in a −80 °C freezer until measurements. Samples were measured using an f80 lens in front of the sample container (Fig. 8). The sample was measured for 3 frames in a disposable cuvette (#7592-00, Sigma-Aldrich, MO, US) with 60 seconds per frame.
